# Usefulness of event-related potentials in the assessment of mild cognitive impairment

**DOI:** 10.1186/1471-2202-9-107

**Published:** 2008-11-05

**Authors:** Vasileios Papaliagkas, Vasileios Kimiskidis, Magda Tsolaki, George Anogianakis

**Affiliations:** 1 Department of Experimental Physiology, Aristotle University of Thessaloniki, Greece; 2 Third Department of Neurology, G Papanikolaou Hospital, Thessaloniki, Greece

## Abstract

**Background:**

The aim of this study was to determine if changes in latencies and amplitudes of the major waves of Auditory Event-Related Potentials (AERP), correlate with memory status of patients with mild cognitive impairment (MCI) and conversion to Alzheimer's disease (AD).

91 patients with MCI (mean ± SD age = 66.6 ± 5.4, MMSE score = 27.7) and 30 age-matched healthy control (AMHC) subjects (mean ± SD age = 68.9 ± 9.9) were studied. 54 patients were re-examined after an average period of 14(± 5.2) months. During this time period 5 patients converted to AD. Between-group differences in latency and amplitude of the major AERP waves (N200, P300 and Slow Wave) were determined. Within each group, correlation coefficients (CC) between these characteristics of the different AERP waves were calculated. Finally, for patients, CCs were determined among each AERP wave and their age and MMSE scores. Confirmatory factor analysis (CFA) was used to examine the underlying structure of waveforms both in the control and the patient groups.

**Results:**

Latencies of all major AERP components were prolonged in patients compared to controls. Patients presented with significantly higher N200 amplitudes, but no significant differences were observed in P300 amplitudes. Significant differences between follow-up and baseline measurements were found for P300 latency (p = 0.009), N200 amplitude (p < 0.001) and P300 amplitude (p = 0.05). MMSE scores of patients did not correlate with latency or amplitude of the AERP components. Moreover, the establishment of a N200 latency cut-off value of 287 ms resulted in a sensitivity of 100% and a specificity of 91% in the prediction of MCI patients that converted to AD.

**Conclusion:**

Although we were not able to establish significant correlations between latencies and amplitudes of N200, P300 and SW and the patients' performance in MMSE, which is a psychometric test for classifying patients suffering from MCI, our results point out that the disorganization of the AERP waveform in MCI patients is a potential basis upon which a neurophysiologic methodology for identifying and "staging" MCI can be sought. We also found that delayed N200 latency not only identifies memory changes better than the MMSE, but also may be a potential predictor of the MCI patients who convert to AD.

## Background

Cognitive event-related potentials (ERPs) have been widely used in the study of dementive disorders, including Alzheimer's disease. Of the major waves observed in the ERPs (N200, P300 and Slow Wave), P300 component corresponds to mental processes such as recognition, categorization of stimuli, expectancy or short-term memory while there are many regions in the brain, especially in the temporal lobe, the parietal lobe and the hippocampus which are thought to be responsible for its generation [1].

The numerous clinical P300 studies [1-6], strongly suggest that this ERP component, elicited by auditory, visual, olfactory or somatosensory stimuli [7], may be clinically useful as an index of cognitive function.

N200 wave, composed of N2a, known as mismatch negativity (MMN) and N2b, may represent short duration memory functions and preattentive storage taking place in sensory cortex 26 [8]. The temporal cortex, the frontal lobe as well as the thalamus and the hippocampus contribute to the generation of MMN [9], whereas the frontal and the superior temporal lobe are also involved in the generation of the N2b wave [10], which is measured in the present study. N200 wave may indicate an early cognitive elaboration concerning subject's attention orientation [11].

The Slow wave (SW) reflects a later, final stage of the stimulus' evaluation and its' relation to P300 remains unclear [12,13]. To our knowledge, there is no published data on the use of this ERP component in the evaluation of MCI or AD.

MCI is described as the transitional stage between normal cognitive changes of aging and the cognitive decline caused by AD. The pathogenesis of MCI is currently unknown.

MCI has recently attracted clinical and research interest, primarily due to the fact that MCI patients are at increased risk for progressing to AD [14]. It is thought that the early identification of those subjects destined to convert to AD may offer the opportunity of therapeutic intervention in the initial stages of the neuropathologic processes leading to dementia, thereby substantially increasing the probability of therapeutic success. Accordingly, the need arises to diagnose and monitor cognitive decline in patients with MCI in an objective manner and to this end a number of electrophysiological techniques have been investigated, including ERPs. Bennys et al. [2] observed that N200 and P300 latencies were significantly prolonged in AD patients when compared to either MCI patients or controls and may be used for categorizing patients into one of the three groups. Golob et al. [3] also studied patients with MCI and AD over a period of 5 years and concluded that P300 latency increased with normal aging and is further prolonged in MCI patients. Except for prolonged P300 latencies, P300 amplitudes were significantly reduced in AD patients when compared to MCI patients or healthy controls [4] or when compared to just healthy age-matched controls [15].

In our own work [16] with 22 MCI patients we had observed that (a) the latencies of N200, P300 and SW increase with age (p < 0.05); (b) the latency of N200 correlates positively (p < 0.05) with that of P300; (c) the latency of the SW correlates positively with the latency (p < 0.01) and the amplitude (p < 0.05) of P300, but not with either the latency or the amplitude of N200; (d) the amplitude of P300 correlates positively (p < 0.01) with the amplitude of SW and (e) the performance of the patients in the MMSE does not correlate with the amplitudes or latencies of N200, P300 and SW.

Although these results are in line with the findings of many studies [2-4,15] the fact remains that there is substantial fragmentation regarding the goals of the different studies reported on MCI, coupled in most cases, with a relative small size for the MCI populations studied (Table 1). In addition, the work of Bennys et al. [2] refers to groups of different mean age each and, in the case of AD, substantially different educational background, while the work of Golob et al. [3] focuses on the P50, N100 and P300. Furthermore, no study so far examined the effects of MCI on the SW.

**Table 1:** ERP studies on MCI patients

**Reference**	**Patients**	**Controls**
[2]	30 AD, 20 MCI	10
[3]	14 AD, 41 MCI	22 young 44 age-matched
[6]	Role of cholinesterase inhibitors, 15 MCI	15
[26]	26 AD, 38 MCI	20
[27]	17 MCI	16
[28]	14 AD, 16 MCI	15
[29]	Olfactory potentials, 14 AD, 8 MCI	8
[4]	30 AD, 26 MCI	26
[5]	15 MCI	12
[30]	14 MCI	14

Accordingly, the aim of the present study was 1) to determine if there are differences in latencies and amplitudes of N200, P300 and SW between patient and control group and also between different age-groups and 2) to identify ERP parameters that could help us to electrophysiologically diagnose MCI. In addition, study participants were clinically and neurophysiologically re-examined so as to explore the evolution of ERPs over time and investigate their predictive value in identifying at an early stage those particular patients that subsequently convert to AD.

## Methods

### Stimuli and Procedures

Auditory event-related potentials were elicited using a simple discrimination task, the so-called oddball paradigm. Event-related potentials use two different tones, an interstimulus interval of several seconds, with the target oddball stimulus presented less frequently than the non-target or standard stimulus. Briefly, a series of binaural tones at 70 dB sound pressure level (SPL) with a 10 ms rise/fall and a 100 ms, plateau time was presented to all subjects. The auditory stimuli were presented in a random sequence with target tones of 2000 Hz occurring 20% of the time and standard tones of 1000 Hz occurring 80% of the time at a rate of 0.5 Hz [17]. The subject is required to distinguish between the two tones by responding to the target (i.e. mentally counting) and not responding to the standard [18]. Patients were instructed to pay attention in distinguishing the tones, count the target tones silently and report the total number at the end of the exam.

EEG activity was recorded (filter bandpass:0.1–50 Hz, analysis time:1 sec) from scalp AgCl electrodes at Cz and Pz sites according to the 10/20 system referred to linked earlobe electrodes, with a right hand ground. Artifacts caused by ocular movements ± 50 μV were automatically rejected. Each patient was tested twice to ensure that waveform components are reproducible. The peak of the ERP components was measured as follows: if the waveform was smooth, the maximal amplitude point was taken as a peak. Otherwise, the leading and trailing slopes of the waveform were extended, and the intersection point was determined [17].

In order to reduce electrode impedance, we used a special type of paste (Elefix Nihon-Kohden, EEG paste Z-401 CE), while the auditory event-related potentials were elicited and analyzed by means of Neuropack 4 (Nihon-Kohden, Tokyo) equipment.

### Subjects

The study group consisted of a total of 91 patients with mild cognitive impairment and 30 age-matched control subjects. MCI was diagnosed according to the widely used criteria of Petersen et al. [14] which include: 1. Memory complaint, preferably corroborated by an informant. 2. Impaired memory function for age and education. 3. Preserved general cognitive function. 4. Intact activities of daily living and 5. Absence of dementia.

Fifty-four patients were re-examined after an average period of 14(± 5.2) months (range from 7 to 26 months). Analyses were conducted on the entire sample and on subgroups of subjects aged ≤ 65 and > 65 years. The basic demographic characteristics of the participant groups are shown in Table 2.

**Table 2:** Demographic and baseline ERP characteristics of the participant groups

**Parameter**	**Patients**	**Controls**	**Statistic**
N (female-male)	91 (56–35)	30(15–15)	χ^2 ^= 0.27
Age Mean (SD)	67.1(6.9)	68.7(9.9)	t = 0.96 p = 0.34
Age range	46–83	49–90	-
Age ≤ 65 (female)	38(28)	11(5)	χ^2 ^= 0.08
Age > 65 (female)	53(28)	19(10)	χ^2 ^= 0.99
N200_L _(mean ± SD), ms	245.1 ± 29.9	234.4 ± 24.3	U = 1215, p = 0.371
P300_L _(mean ± SD), ms	399.7 ± 43.3	367.6 ± 28.9	U = 783, p < 0.001
SW_L _(mean ± SD), ms	534.1 ± 60.6	495.0 ± 45.6	U = 800, p = 0.001
N200_A _(mean ± SD), μV	9.3 ± 0.44	7.3 ± 0.39	U = 897, p = 0.025
P300_A _(mean ± SD), μV	16.2 ± 0.77	13.5 ± 0.57	U = 1032, p = 0.107

The patients were recruited from the Third Neurological Clinic of the Aristotle University of Thessaloniki in Papanikolaou General Hospital. The study was approved by the Ethics Committee of the Aristotle University of Thessaloniki and it was performed according to the declaration of Helsinki. An informed consent was obtained from all patients, before they were admitted to the study. The patients and the controls were assessed with neuropsychological tests, which include the Mini Mental State Examination (MMSE) [19,20] and by auditory event-related potentials. The average performance of the patients in the MMSE test was 27.7/30, whereas all control subjects scored 29.7/30.

### Statistical analysis

Statistical analysis was performed with SPSS 15.0 for Windows (SPSS Inc) and AMOS 7.0 for the confirmatory factor analysis.

Of the measured parameters, age and the latencies of control subjects were found to be normally distributed (Shapiro-Wilk test).

Univariate ANOVA was used to determine differences in ERP parameters between MCI patients and controls. A confirmatory factor analysis was also used to examine the underlying structure of the waveform both in the control and the patient groups. Chi-square test was used to compare frequencies between groups.

Pearson's correlation coefficient (r) was computed when the variables had normal distribution, whereas Spearman's r_s _was used when at least one variable did not follow the normal distribution.

Unpaired Student's t-test was performed to compare the ages of patients and controls. Changes between baseline and follow-up measurements were evaluated using the Wilcoxon matched-pairs sign-rank test. The Mann-Whitney U-test was used for comparisons between patients and controls.

Probability values < 0.05 (two-tailed) were considered statistically significant.

## Results

### Performance

**Figure 1:**
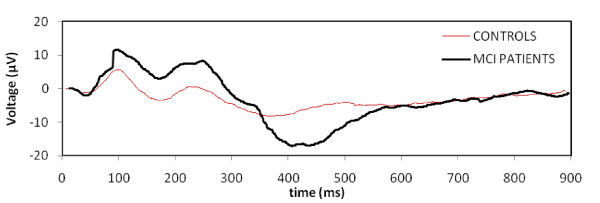
Grand average AERP waveforms for MCI patients (n = 91) and controls (n = 30).

All subjects responded to the target tones of the auditory event-related potentials with an accuracy of over 95%. Grand average AERP waveforms for MCI patients (n = 91) and controls (n = 30) are given in Figure 1.

### Correlation between major ERP components at baseline exam

Comparisons were made for AERPs at baseline exam in patients and controls to evaluate the relation between age, latencies and amplitudes. The means (standard deviations) of the AERP characteristics at baseline of the two groups are shown in Table 2.

#### a. Patient group

**Figure 2:**
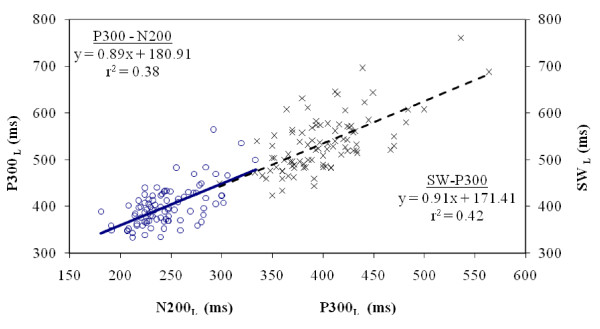
Regression lines of P300-N200 and SW-P300 latencies in MCI patients.

**Figure 3:**
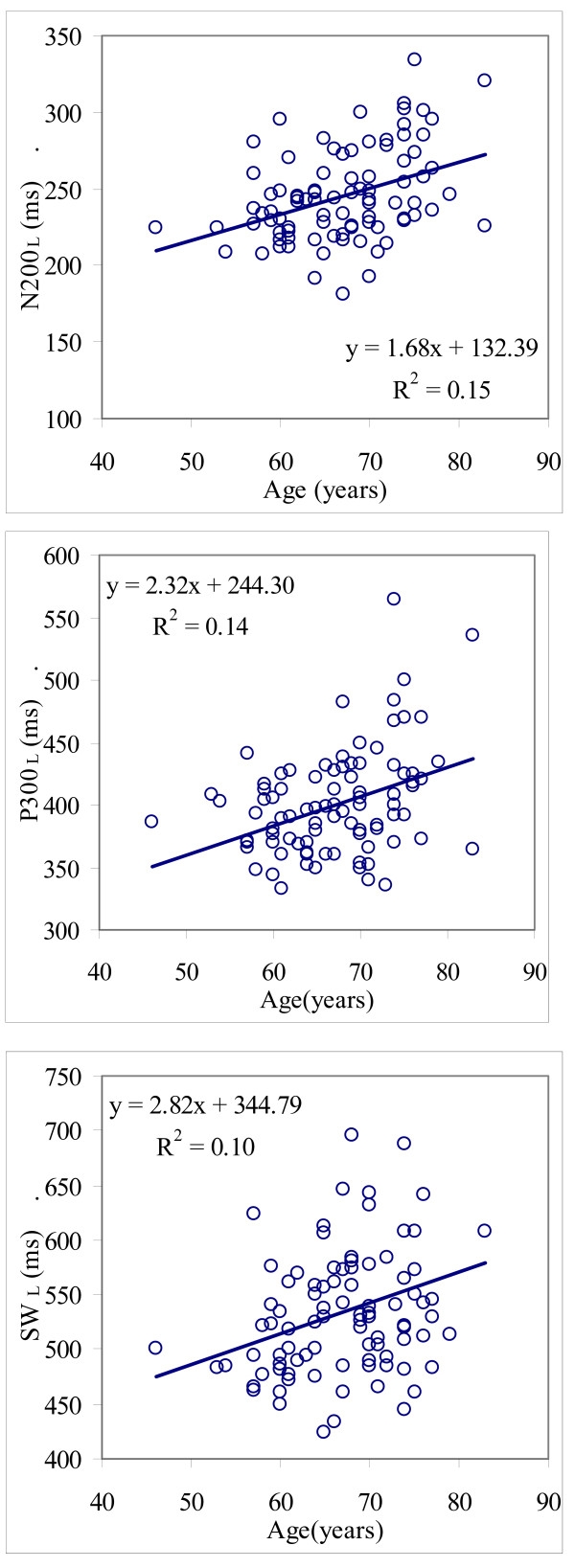
**Scattergramms showing the relation between AERP latencies and age in MCI patients.** From top to bottom: N200_L_-age, P300_L_-age and SW_L_-age.

In the 91 patients we studied at baseline the results reveal that:

(a) The latency of N200 correlates highly significantly (r_s _= 0.55, p < 0.001) with that of P300.

(b) The latency of the P300 wave has a highly significant statistical correlation with the latency of the SW (r_s _= 0.59, p < 0.001). The correlation between N200 and SW latencies is also significant (r_s _= 0.26, p < 0.05). Therefore there seems to be a relationship between the latencies of all three major waves of AERPs and each one is dependant upon the other. Fig. 2 shows the regression lines between P300-N200 and SW-P300 latencies. It is evident that the equations and the correlation coefficients of the two regression lines are practically identical and therefore the value of SW can be determined by extrapolation of the P300-N200 latencies regression line.

(c) There is a highly significant association (p < 0.001) between P300-N200 latency difference and P300 (r_s _= 0.69) and SW (r_s _= 0.50) latencies. Moreover P300-N200 latency difference is significantly correlated with P300 amplitude (r_s _= 0.24, p < 0.05).

(d) The difference between SW and P300 latencies is highly correlated with the difference between the latencies of SW and N200 (r_s _= 0.78, p < 0.001).

(e) Significant correlation was found between N200 and P300 amplitudes (r_s _= 0.33, p < 0.01).

(f) Statistically significant positive correlations were found between N200, P300 and SW latencies and age (N200 r_s _= 0.37 p < 0.001; P300 r_s _= 0.33, p < 0.01 and SW r_s _= 0.27 p < 0.01) and a negative correlation between P300 amplitude and age (r_s _= 0.26, p < 0.05). The regression lines between latencies of ERPs and age are presented in Figure 3.

#### b. Control group

In the 30 healthy control subjects we studied the results show that:

(a) Latencies of N200, P300 and SW increase with age (p < 0.01) (For N200 r = 0.52, for P300 r = 0.44, for SW r = 0.40).

(b) Highly significant correlations were observed between the latencies of N200 and P300 (r = 0.58, p < 0.001) and between P300 and SW latencies (r = 0.80, p < 0.001). Significant correlation is observed also between N200 and SW latencies (r = 0.40, p < 0.05)

(d) The latency difference P300-N200 is highly associated with P300 latency (r = 0.59, p < 0.001).

(e) As in patients, the difference between SW and P300 latencies is highly correlated with the difference between the latencies of SW and N200 (r = 0.88, p < 0.001). Moreover it is highly related with SW wave latency (r = 0.86, p < 0.001) and associated with P300 latency (r = 0.46, p < 0.01).

(f) Similarly to the observation made in the patients group, the amplitudes of the major waves in healthy controls decrease with age. This decrease is highly significant for P300 wave (r = -0.60, p < 0.001) but not for N200 wave (r = -0.22, p < 0.5).

(g) There seems to be a highly significant correlation between N200 and P300 amplitudes (r = 0.69, p < 0.001).

### Correlation of ERP latencies and amplitudes in MCI patients with MMSE performance

No correlation was observed between latencies and amplitudes of N200, P300 and SW and the patients' performance in MMSE.

### Group differences (MCI/controls)

The Mann-Whitney test was used for intergroup comparisons of the ERP characteristics. P300 and SW latency were significantly prolonged in patients compared to the control group (for P300 U = 783, p < 0.001; for SW U = 800, p = 0.001). On the other hand no difference was observed in N200 latencies (U = 1215, p = 0.367). Moreover, patients had significantly higher N200 amplitude (U = 897, p = 0.025) than controls, whereas P300 amplitude did not seem to differ between the two groups (U = 1032, p = 0.107). The statistical significances of the intergroup differences are shown in Table 2.

In order to test the AERP waveform difference in MCI patients compared to controls, confirmatory factor analysis (CFA) was employed to examine the underlying structure of the waveform both in the control and the patient groups. Given that the sample size of the present study was relatively small for such an analysis, bootstrapping techniques were used. These techniques are regarded an ideal means to tackle problems in situations where the assumption of adequate sample size is not met [21].

A one-factor model was postulated and tested for the five measured variables, which were the latencies of N200, P300 and SW (N200_L_, P300_L _and SW_L_) and the amplitudes of N200 and P300 (N200_A _and P300_A_). All variables were transformed to z-scores to share the same scale. Initial analyses showed that P300 latency had negative error variances for both groups and thus it was excluded from the model.

**Figure 4:**
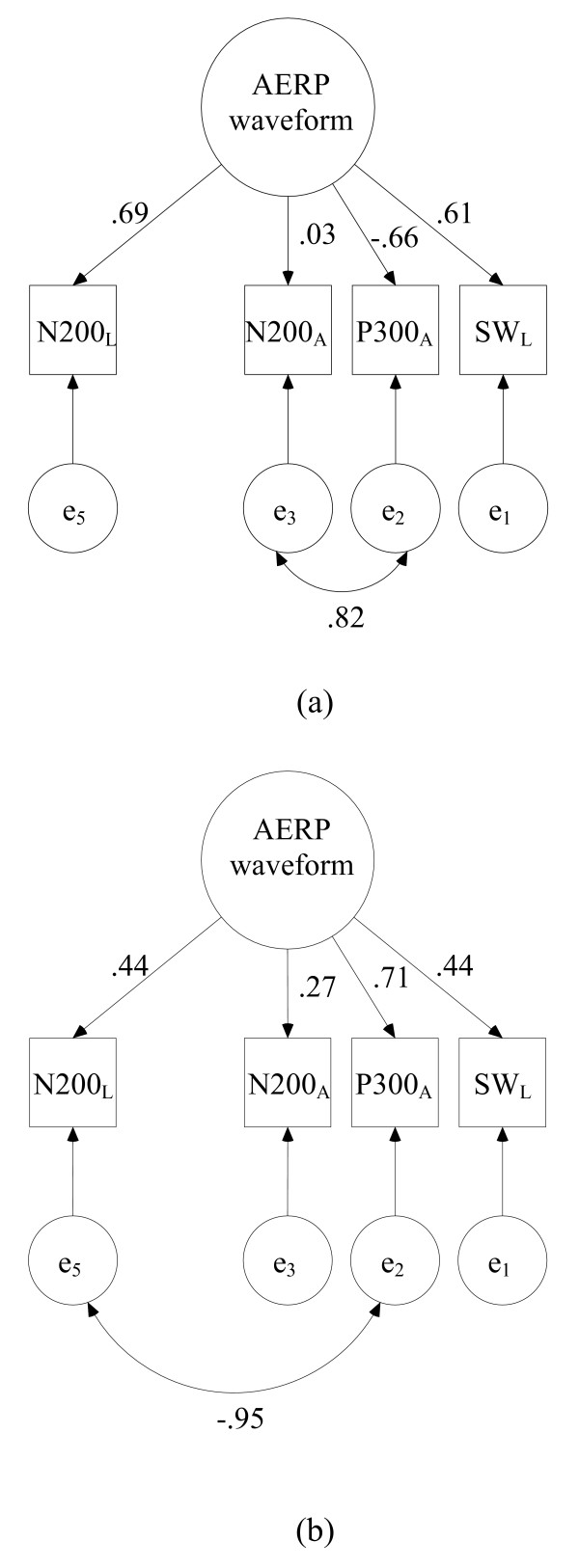
Confirmatory factor analysis results of the ERP waveform for (a) control and (b) patients group.

Figures 4a and 4b depict the AERP final models for the two groups. All fit indices suggested a good fit of the models to the data: For the control group, χ^2 ^= 3.45, df = 2, p = 0.178, CFI = 0.954, SRMR = 0.086 and for the MCI patients group, χ^2 ^= 3.68, df = 2, p = 0.159, CFI = 0.935, SRMR = 0.062. As suggested by the modification indices, to improve the fit of the models, an error covariance between N200_A _and P300_A _was added in the case of the control group and between N200_L _and P300_A _in the case of the patient group.

As shown by the fit indices, both models had acceptable fit to the data. However, substantial differences between them can be observed. In particular, the latencies of N200 and SW had higher factor loadings for the control group (0.69 and 0.61) than the patients group (0.44 for both variables), implying that MCI reduces the contribution of these two components on the entire ERP waveform. Also there is a substantial difference in the factor loadings for the amplitudes of N200 and P300 in the two groups (0.03 and 0.27 for N200_A _and -0.66 and 0.71 for P300_A_).

### Analysis of Variance (ANOVA)

A univariate ANOVA was conducted for a more comprehensive study of the relation between AERP parameters (Table 3). Each variable was obtained as a dependent variable with group (MCI patients/AMHC) and age-group as fixed variables. When each ERP component was examined independently, differences between patient and control group were found for P300 and SW latencies (p < 0.001) and N200 amplitude (p = 0.029), whereas between the two age-groups the differences were highly significant (p < 0.001) for N200_L_, P300_L _and SW_L_, significant (p = 0.005) for P300_A _and non-significant (p = 0.065) for N200_A_.

**Table 3:** Results of univariate ANOVA of basic ERP components (Statistically significant differences are shown in bold)

**ERP Component**	**Controls**			**Patients**		**Significance**
	
	***Age-group***	***Mean***	***S.E.***	***Mean***	***S.E.***	***p***
N200_L_	≤ 65	220.7	8.19	235.3	4.41	0.064
	> 65	244.6	6.23	252.1	3.73	
P300_L_	≤ 65	353.6	11.54	381.4	6.21	**< 0.001**
	> 65	379.9	8.78	412.8	5.26	
SW_L_	≤ 65	475.0	16.68	515.5	8.97	**< 0.001**
	> 65	506.4	13.04	547.5	7.60	
N200_A_	≤ 65	8.05	1.27	10.62	0.69	**0.029**
	> 65	6.80	1.03	8.37	0.59	
P300_A_	≤ 65	17.02	2.15	17.80	1.17	0.114
	> 65	10.85	1.68	15.08	0.99	
P300_L_	≤ 65	371.1	9.88	387.3	5.22	**0.003**
(covariate N200_L_)	> 65	378.5	7.29	405.4	4.48	
SW_L_	≤ 65	485.3	16.80	519.0	8.88	**0.004**
(covariate N200_L_)	> 65	505.6	12.75	543.2	7.62	
SW_L_	≤ 65	510.5	13.56	525.2	7.05	0. 288
(covariate P300_L_)	> 65	521.4	10.26	528.1	6.30	
P300_A_	≤ 65	17.02	2.15	17.80	1.17	0.341
(covariate N200_A_)	> 65	10.85	1.68	15.08	0.99	

The effect of earlier ERP components on the later ones was studied using preceding components as covariates. When N200_L _was used as a covariate for the analysis of P300_L_, a significant difference (p = 0.003) between groups, but not between age-groups (p = 0.082) was found. In the analysis of SW latency with N200_L _as a covariate, only the between-groups comparison was found to be significant (p = 0.004), whereas with P300_L _as a covariate, no difference between groups or age-groups was found (p = 0.288 and 0.487 respectively). This indicates that P300 and SW latencies carry the same information, a finding that is in agreement with the regression lines shown in Figure 2.

In the analysis of P300 amplitude with N200 amplitude as a covariate, the significance in the difference between the two age-groups remained (p = 0.024).

### Change of ERP components with time

Significant differences between follow-up and baseline measurements were found for P300_L _(p = 0.009), N200_A _(p < 0.001) and P300_A _(p = 0.05) (Wilcoxon matched-pairs sign-rank test).

Comparison of differences in ERP parameters between the patients who during the follow-up converted to AD and those who remained stable (Table 4), revealed that N200 latency was the only parameter with statistically significant difference (Mann-Whitney test, Z = -3.58, p < 0.001). In the MMSE test the difference between these two sub-groups showed a trend towards significance (Z = -1.858, p = 0.063), indicating that N200_L _measurements at baseline not only identify memory changes better than the MMSE, but also may serve as a potential predictor of the MCI patients who convert to AD. In particular the use of a cut-off value of 287 ms resulted in an AUC of 0.988 (95% CI = 0.939–0.998, p < 0.001), a sensitivity of 100% (95% CI = 48–100%) and a specificity of 94% (95% CI = 86.9–98.1%).

**Table 4:** Comparison of ERP parameters between MCI stable and AD-converting subgroups (Statistically significant differences are shown in bold)

**Parameter**	**Total**	**MCI stable**	**AD-converters**	**Statistic**	**p**
N (female)	91(56)	86(54)	5(2)	χ^2 ^= 1.037	0.309
Age Mean (SD)	67.1(6.9)	66.8(6.7)	72.6(9.5)	Z = -1.666	0.096
MMSE Median	28	28	26	Z = -1.858	0.063
(IQR)	(26–29)	(26–29)	(24–28)		
N200_L _median	240	238.5	306	Z = -3.580	**< 0.001**
(IQR), ms	(225–265)	(224–258)	(291–327)		
P300_L _median	394	394	467	Z = -1.916	0.055
(IQR), ms	(370–424)	(370–422)	(387–518)		
SW_L _median	527	528	522	Z = -0.165	0.869
(IQR), ms	(488–571)	(490–570)	(474–684)		
N200_A _median	8.75	8.41	11.25	Z = -1.734	0.083
(IQR), μV	(6.25–11.25)	(6.25–11.25)	(8.82–16.22)		
P300_A _median	13.90	13.95	13.75	Z = -0.944	0.345
(IQR), μV	(10.45–19.65)	(10.50–20.0)	(9.40–15.25)		

There was a significant difference in N200 latency medians between controls, MCI stable patients and AD-converters (Kruskal-Wallis Test, p = 0.001). The difference was significant between controls and AD-converters (Mann-Whitney test, p = 0.001), but not between controls and MCI stable patients (p = 0.632).

## Discussion

In this study AERPs were conducted to determine if changes in latencies and amplitudes of the major waves of AERPs, correlate with memory status of patients with MCI and conversion to AD.

As noted, patients with MCI are at high risk for developing Alzheimer's disease [22] and according to Petersen et al. [23], they convert to Alzheimer's disease at a rate of about 12% per year and by the end of the sixth year, after MCI is diagnosed, 80% of patients with MCI convert to Alzheimer's disease. In another study [22] over a period of five years, less than 50% of patients with MCI converted to AD. Therefore, early diagnosis in these patients is very important, and for this purpose ERPs have been employed in several studies. In a study involving 12 healthy elderly controls and 15 MCI patients, auditory event-related potentials following the "oddball" paradigm were used [24]. The median performance of patients with MCI in the MMSE was 27.7. P300 latency was significantly delayed in MCI patients, but N200 latency as well as the amplitudes of the two components were found not to be statistically different between the two groups. Pokryszko-Dragan et al. [25], also observed that in patients with mild and moderate Alzheimer's disease, P300 latency was significantly prolonged compared to healthy subjects. From the characteristics of the P300 wave (latency, amplitude), which we studied, P300 amplitude was also found not to be significantly different between patient and control group, whereas P300 latency was significantly delayed in patients compared to control subjects.

Our results confirm those presented by Golob et al. [5]. However, having also studied the N200 and SW characteristics, we also observed that SW latencies are significantly delayed in MCI patients when compared to healthy controls. In addition, we observed that the strong correlation which exists (a) between the N200-to-SW latency difference and the latencies of P300 or SW and (b) between the P300-to-SW latency difference and the latencies of P300 or SW, in control subjects, disappears in MCI patients. Another observation is that, both the amplitudes of P300 and N200-to-P300 latency difference correlate with the latency of SW in MCI.

We interpret this finding as a disorganization of the AERP waveform in MCI patients whereby the generators of the N200, P300 and SW of the AERP are "decoupled" in MCI. In this respect we also observed that (a) the strong correlation that exists between N200 and P300 amplitudes in our control subjects disappears in the case of MCI; (b) the P300 amplitude becomes strongly correlated with the N200-to-P300 latency difference and (c) Age stops being correlated with P300 amplitude. In our view therefore, while, in AMHC, N200 wave can be considered as the "initiator" of both the P300 and the SW, in MCI it does not influence the generation of SW. The observation that in MCI the N200-to-P300 latency difference does not correlate with either the N200-to-SW latency difference or the P300-to-SW latency difference, further underscores this point. Further evidence of the disorganization of the AERP waveforms and the "decoupling" of the wave generators in MCI patients compared to healthy controls is provided by the results of the CFA, where substantially different loading factors of the AERP parameters of the two groups were found for the two groups.

According to the ANOVA analysis of our data, the between age-groups (≥ 65 and > 65 years) difference observed in P300 latency is due to N200 latency, while the between groups difference which is observed in SW latency is due to P300 latency. This means that, P300 and SW may carry the same information and be due to the same generator, a finding that comes to agreement with Garcia- Larrea and Cezanne-Bert [31], but contrasts with the conclusions of Freidman et al [13].

Differences between patient and control group were found for P300 and SW latencies (p < 0.001) and N200 amplitude (p < 0.05), whereas differences between the two age-groups were found for all components except for N200 amplitude.

The fact that MMSE does not correlate with the amplitudes or latencies of N200, N300 and SW, indicates that the latencies of these major ERP components, when viewed as statistical functions of age, are orthogonal to the corresponding set of MMSE scores [32], which in turn, implies that these latencies can be used as measures of MCI independent of the MMSE score.

Finally, from our follow up study it is also evident, that N200 latency is capable of predicting which MCI patients will convert to AD and therefore seems to have a predictive value for AD diagnosis. The choice of a cut-off value of 287 ms resulted in a sensitivity of 100% and specificity of 91%. Moreover N200 latency was significantly higher in the 5 MCI patients that converted to AD compared to MCI stable patients (p < 0.001). The mean age of these 5 AD-converters (72.6 years) was higher than that of the MCI stable patients (66.8 years), however, this difference was not significant (p = 0.096, Table 4).

It must be emphasized that the small number of the MCI patients who converted to AD does not allow for a general conclusion to be drawn. However, this finding is consistent with results presented in other studies [2,33], regarding the usefulness of N200 latency measurements in predicting MCI conversion to AD.

## Conclusion

Despite the fact that in our study we measured latencies and amplitudes of all the major waves of the AERPs (N200, P300 and SW) in the largest number of MCI patients to date, we like to point out that we were not able to establish significant correlations between latencies and amplitudes of N200, P300 and SW and the patients' performance in MMSE, which is a basic psychometric test for the assessment of patients suffering from MCI. This conclusion, we believe, is in line with the conclusions of the majority of the relevant known studies (Table 1), which also fail to demonstrate that the performance of MCI patients in MMSE correlates with ERP parameters. In addition, our results point out to the disorganization of the AERP waveform in MCI patients (whereby the generators of the N200, P300 and SW of the AERP are "decoupled" in MCI) as a potential basis upon which a neurophysiologic methodology for identifying and "staging" MCI can be sought. The significant correlations that exist between the different peaks of the AERP waveform in AMHC, which in many ways break down in MCI, can be used for this purpose. This in turn, implies that statistical descriptions of the ensemble of an individual's AERP waveforms are needed for this purpose, rather than the simple averaged AERP waveforms. We have made this the next step in our search for a neurophysiologic indicator of MCI. Valuable aid in this search gives us the observation that N200 latency might serve as a potential useful marker in the early diagnosis of AD.

## Authors' contributions

VP participated in the design of the study, carried out the event-related potentials, performed the statistical analysis and drafted the manuscript. VK helped in the event-related potentials, participated in the design of the study and helped to draft the manuscript. MT recruited the patients and the control subjects and participated in the design of the study. GA participated in the design and coordination of the study and contributed to the preparation of the draft. All authors read and approved the final manuscript.
